# Anti-CD20 as the B-Cell Targeting Agent in a Combined Therapy to Modulate Anti-Factor VIII Immune Responses in Hemophilia a Inhibitor Mice

**DOI:** 10.3389/fimmu.2013.00502

**Published:** 2014-01-06

**Authors:** Chao Lien Liu, Peiqing Ye, Jacqueline Lin, Chérie L. Butts, Carol H. Miao

**Affiliations:** ^1^Center for Immunity and Immunotherapies, Seattle Children’s Research Institute, Seattle, WA, USA; ^2^School of Medical Laboratory Science and Biotechnology, College of Medical Science and Technology, Taipei Medical University, Taipei, Taiwan; ^3^Biogen Idec, Inc., Weston, MA, USA; ^4^Department of Pediatrics, University of Washington, Seattle, WA, USA

**Keywords:** anti-CD20, factor VIII, hemophilia, tolerance induction, immunomodulation, B-cell depletion

## Abstract

Neutralizing antibody formation against transgene products can represent a major complication following gene therapy with treatment of genetic diseases, such as hemophilia A. Although successful approaches have been developed to prevent the formation of anti-factor VIII (FVIII) antibodies, innovative strategies to overcome pre-existing anti-FVIII immune responses in FVIII-primed subjects are still lacking. Anti-FVIII neutralizing antibodies circulate for long periods in part due to persistence of memory B-cells. Anti-CD20 targets a variety of B-cells (pre-B-cells to mature/memory cells); therefore, we investigated the impact of B-cell depletion on anti-FVIII immune responses in hemophilia A mice using anti-CD20 combined with regulatory T (T_reg_) cell expansion using IL-2/IL-2mAb complexes plus rapamycin. We found that anti-CD20 alone can partially modulate anti-FVIII immune responses in both unprimed and FVIII-primed hemophilia A mice. Moreover, in mice treated with anti-CD20+IL-2/IL-2mAb complexes+rapamycin+FVIII, anti-FVIII antibody titers were significantly reduced in comparison to mice treated with regimens targeting only B or T cells. In addition, titers remained low after a second challenge with *FVIII* plasmid. T_reg_ cells and activation markers were transiently and significantly increased in the groups treated with IL-2/IL-2mAb complexes; however, significant B-cell depletion was obtained in anti-CD20-treated groups. Importantly, both FVIII-specific antibody-secreting cells and memory B-cells were significantly reduced in mice treated with combination therapy. This study demonstrates that a combination regimen is highly promising as a treatment option for modulating anti-FVIII antibodies and facilitating induction of long-term tolerance to FVIII in hemophilia A mice.

## Introduction

Hemophilia A is an X-linked, congenital bleeding disorder resulting from a deficiency of factor VIII (FVIII). Approximately 35% of patients with hemophilia A develop complications of anti-FVIII neutralizing antibodies following FVIII protein replacement therapy (1, 2). In order to overcome anti-FVIII immune responses, we sought transient immunosuppressive strategies that can reduce pre-existing antibodies and induce long-term tolerance to FVIII. CD20 is a 35-kDa transmembrane protein expressed on B-cells from the pre-B-cell stage to mature B lymphocytes, but not plasma cells (3). Monoclonal antibodies (mAbs) against human CD20 (Rituximab) induce rapid B-cell depletion (4) and is currently approved by the Food and Drug Administration (FDA) for treatment of non-Hodgkin B-cell lymphomas (5, 6) and several autoimmune disorders including type 1 diabetes (T1D) (7), rheumatoid arthritis (8), and Sjögren’s syndrome (SS) (9). Anti-CD20 depletes B-cells via several mechanisms (10, 11), such as direct induction of apoptosis, antibody-dependent cell-mediated cytotoxicity (ADCC) (12), and complement-dependent lysis (CDC) (13), which are considered to be immediate and comparatively short-acting. Nevertheless, the clinical response to a single course of the anti-CD20 mAb can be late acting and prolonged. This has led to the suggestion that anti-CD20 could also have an immunization effect (14); however, it is unknown whether this correlates with clinical outcome. Recently anti-CD20 IgG_1_ (15) and IgG_2a_ (16) molecules have been used successfully to prevent the production of anti-FVIII antibodies.

Webster et al. (17) defined a strategy in which complexes of IL-2/IL-2-specific mAbs (JES6-1A12) can be used to selectively expand CD4^+^CD25^+^Foxp3^+^ regulatory T (T_reg_) cells *in vivo* with little or no change in other cell populations. This approach has been used to successfully treat asthma (18) and experimental myasthenia gravis (MG) (19) in mouse models. In addition, rapamycin is currently used as an immunosuppressive agent to prevent acute graft rejection in humans (20). Rapamycin combines with the intracellular immunophilin FK506-binding protein (FKBP12) to form FKBP12-rapamycin complexes that inhibit the activity of mammalian target of rapamycin (mTOR) and result in inhibiting effector T-cell (T_eff_) proliferation (21). Rapamycin not only increased T_reg_:T_eff_ cell ratios but also improved the suppressive activity of T_reg_ cells (22, 23).

In our previous studies, administration of IL-2/IL-2mAb complexes prevented anti-FVIII immune responses in hemophilia A mice following gene or protein replacement therapy (24, 25). Nevertheless, overcoming pre-existing antibody responses in primed subjects remains challenging. Anti-FVIII neutralizing antibodies persist in part due to memory B-cells (26). Moreover, molecular studies have shown that long-lived plasma cells (LLPCs) can support chronic inflammatory processes by secreting pathogenic antibodies for long periods (27, 28). It is hypothesized that LLPCs may also play an important role in prolonged production of anti-FVIII antibodies in hemophilia A patients. In this study, we developed a treatment strategy of single or combination therapy using agents targeting B-cells (to eliminate memory responses) and those inducing T_reg_ cell expansion (to suppress T helper cell function). By using a combination of anti-CD20+IL-2/IL-2mAb complexes+rapamycin, anti-FVIII immune responses were significantly reduced. Hemophilia A mice treated with combination therapy showed little or no anti-FVIII antibodies titers, and this was also evident after a second challenge with *FVIII* plasmid. This study sought to identify strategies toward induction of immune tolerance to FVIII transgene product following gene therapy and to demonstrate that combination therapy targeting B and T lymphocytes can be a viable option.

## Results

### Anti-CD20 treatment can regulate anti-FVIII production in a non-viral gene therapy model

**Figure 1:**
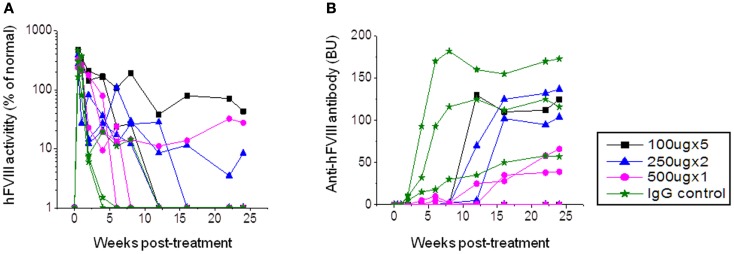
***Factor VIII* gene expression and anti-FVIII antibody formation after *FVIII* plasmid+anti-CD20 treatment in hemophilia A mice**. Four groups of hemophilia A mice were treated with *FVIII* plasmid (50 μg/treatment/mouse) at day 0 and i.v. injection of anti-CD20 at various doses and schedules as listed in the following: group 1: 100 μg/treatment/mouse, injected at days −2, 0, 3, 6, and 9. Group 2: 250 μg/treatment/mouse, injected at days 0 and 14. Group 3: 500 μg/treatment/mouse, injected at day 0. Group 4: control rat IgG, 250 μg/treatment/mouse, injected at days 0 and 14. Peripheral blood samples were collected at different time points to examine FVIII activities **(A)** and inhibitor titers **(B)**. Each symbol represents data obtained from an individual mouse. Data shown is representative of two independent experiments.

To test if B-cell depletion can regulate anti-FVIII immune responses, we utilized anti-CD20 IgG2a antibody (anti-CD20) in a murine model. Hemophilia A mice were divided into two treatment groups (Figure 1 and Figure S1 in Supplementary Material): *FVIII* plasmid-treated mice were given anti-CD20 (250 μg/mouse) on days 0 and 14 combined with a *FVIII* plasmid (pBS-HCRHPI-FVIIIA; 50 μg/mouse) expressing B domain-deleted hFVIII under the control of the liver-specific hAAT promoter (HP) and the hepatic control region (HCR) on day 0. Control mice were treated with rat IgG2a (250 μg/mouse) on days 0 and 14. Anti-CD20 significantly reduced total B220^+^/CD19^+^ B-cells (80–90% reduction) both in blood (Figures S1A,B in Supplementary Material) and spleen (Figure S1C in Supplementary Material). B-cell depletion was sustained over 4–6 weeks with gradual return to normal levels at 8 weeks following treatment. No reduction in B-cell levels were observed in IgG2a isotype-treated control and naive mice.

In order to investigate the best treatment schedule and therapeutic effects of anti-CD20 treatment, three groups of hemophilia A mice were injected with three different dosages: 100 μg/mouse (on days −2, 0, 3, 6, and 9), 250 μg/mouse (on days 0 and 14), and 500 μg/mouse (on day 0). Control mice were treated with rat IgG2a 250 μg/mouse on days 0 and 14. All mice were injected with *FVIII* plasmid (50 μg/mouse) at day 0. Following treatment, FVIII activities and neutralizing antibody titers were assessed by aPTT and Bethesda assays at different time points. In the rat IgG2a control group, anti-FVIII antibody appeared within 2 weeks post plasmid injection, increased to high-titers at 3–4 weeks, and maintained high-titer levels through 24 weeks. In addition, initially high levels of FVIII activity decreased to low-undetectable levels within 4 weeks (Figure 1). In the anti-CD20-treated groups, one mouse from each group of mice had persistent FVIII activity without detectable inhibitory anti-FVIII antibodies (Figure 1). The remaining mice displayed delayed immune responses; however, all mice generated moderate to high-titers of neutralizing antibodies with FVIII activity decreasing to undetectable levels at 6–15 weeks. While antibody titers were clearly reduced following anti-CD20 treatment, these titers increased over time. Although mice treated with anti-CD20 were not completely resistant to FVIII immune responses, they all exhibited partial modulatory effects compared to the rat IgG2a treated control mice.

**Figure 2:**
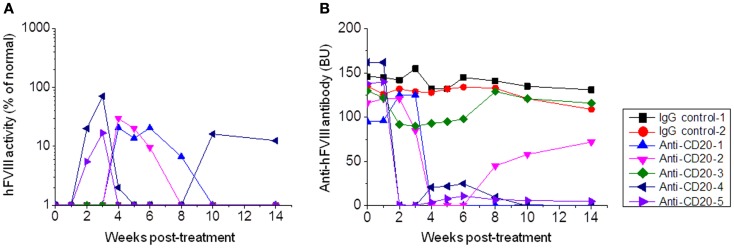
***Factor VIII* gene expression and anti-FVIII antibody titers following anti-CD20 treatment in *FVIII* plasmid-primed hemophilia A mice with pre-existing inhibitors**. Mice were primed with *FVIII* plasmid to induce high-titer inhibitory antibodies at 8 weeks before anti-CD20 treatment. The inhibitor mice were then treated with anti-CD20. Group 1: control rat IgG, 250 μg/treatment/mouse, injected at days −7, −4, and 0. Group 2: anti-CD20, 250 μg/treatment/mouse, injected at days −7, −4, and 0. Peripheral blood samples were collected at different time points to evaluate FVIII activities **(A)** and inhibitor titers **(B)**. Each symbol represents data obtained from an individual mouse. Data shown is representative of two independent experiments.

A similar treatment was given to *FVIII* plasmid-primed hemophilia A mice with pre-existing neutralizing antibodies. These mice were developed by hydrodynamic injection of 50 μg of *FVIII* plasmid via tail vein, and only mice with neutralizing antibody titers >30 Bethesda units (BU) were used. Mice were then treated with anti-CD20 (*n* = 5; 250 μg/mouse) on days −7, −4, and 0. Control mice were treated with IgG2a (*n* = 2; 250 μg/mouse) on days −7, −4, and 0. Plasma samples were collected on day 1 following treatment. As shown in Figure 2, neutralizing antibody titers were maintained at high levels in rat IgG2a control-treated mice. In contrast, neutralizing antibody titers were significantly reduced following anti-CD20 treatment in 80% (four of five) treated mice. In particular, 20% (one of five) of anti-CD20-treated mice showed therapeutic FVIII activities levels for 14 weeks (Figure 2B). These results indicate that anti-CD20 can partially modulate anti-FVIII immune responses both in the FVIII unprimed and primed hemophilia A mice.

### Combination treatment with IL-2/IL-2mAb complexes, rapamycin, and anti-CD20 enhanced *FVIII* plasmid-mediated gene therapy in hemophilia a mice

**Figure 3:**
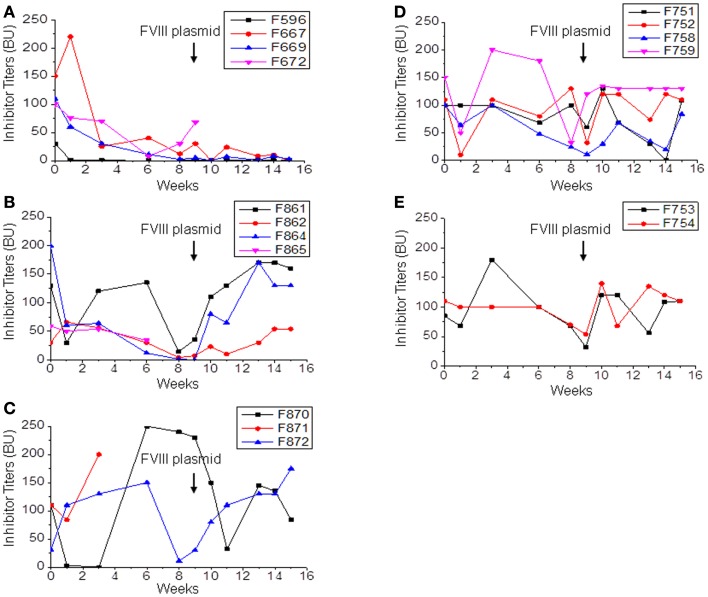
**Immunomodulation with separate or combined therapy by IL-2/IL-2mAb complexes, rapamycin, and anti-CD20 in *FVIII* plasmid-primed hemophilia A mice with pre-existing inhibitors**. Four groups of hemophilia A inhibitor mice were treated separately with different combined regimens: **(A)** IL-2/IL-2mAb complexes+rapamycin+ anti-CD20+FVIII injection (*n* = 4, group 1), **(B)** IL-2/IL-2mAb complexes+anti-CD20+FVIII (*n* = 4, group 2), **(C)** IL-2/IL-2mAb complexes+rapamycin+FVIII injection (*n* = 3, group 3), **(D)** anti-CD20+FVIII injection (*n* = 4, group 4), and **(E)** Control inhibitor mice (*n* = 2, group 5). Each experimental group was treated with indicated immunomodulation regimen weekly for 4 weeks. Anti-FVIII antibody titers were assessed by Bethesda assay over time. Each symbol represents data obtained from an individual mouse. Data shown is representative of three independent experiments.

Since anti-CD20 treatment can partially modulate anti-FVIII immune responses in hemophilia A mice, we investigated whether a combination therapy using anti-CD20 to deplete B-cells and IL-2/IL-2mAb complexes to expand T_reg_ cells (24) can more consistently reduce anti-FVIII responses. Hemophilia A mice were treated with: IL-2/IL-2mAb complexes+rapamycin+anti-CD20+FVIII (*n* = 4, group 1; Figure 3A); IL-2/IL-2mAb complexes+anti-CD20+FVIII (*n* = 4, group 2; Figure 3B); IL-2/IL-2mAb complexes+rapamycin+FVIII (*n* = 3, group 3; Figure 3C); anti-CD20+FVIII (*n* = 4, group 4; Figure 3D); and mock agents (control inhibitor mice; *n* = 2, group 5; Figure 3E) weekly for 4 weeks. FVIII protein (1 U/mouse) was given weekly for 4 weeks for induction of FVIII-specific tolerance during the treatment period. *FVIII* plasmid second challenge was applied at 5 weeks following 4 weeks of treatment (week 9). Except for control mice, all treated groups showed decreased antibody titers. The most significant and prolonged reduction of neutralizing antibody titers was observed using the combination treatment of IL-2/IL-2mAb complexes+anti-CD20+rapamycin+FVIII (Figure 3A). Neutralizing antibody titers were reduced to 0 in 50% (two of four) treated mice, and a reversion of 8% FVIII gene expression was observed. Additional animals (*n* = 3–5/group) have been treated in repeated experiments with similar results to those shown (Figures 3A–E). In addition, treated mice were challenged with non-specific antigen, TNP-ficoll (24), at 16 weeks following treatment (week 20). These animals responded similarly to control/naive mice, demonstrating the tolerance effect was FVIII-antigen-specific.

### Effects on T/B-cell responses in peripheral blood and spleen were significant in treated hemophilia a mice

**Figure 4:**
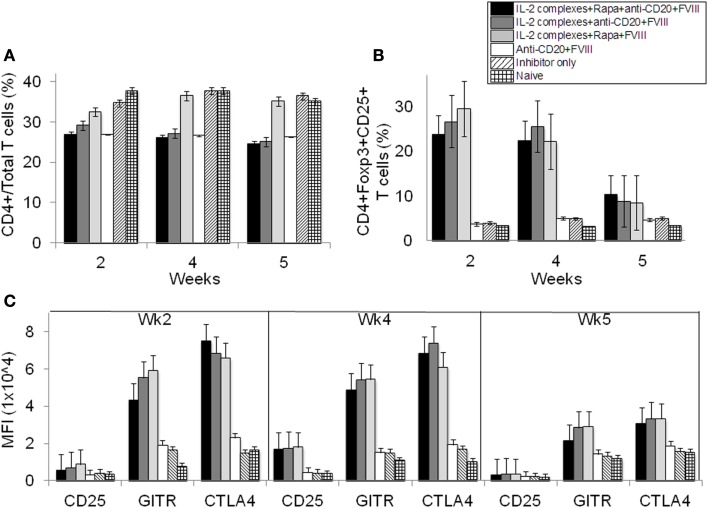
**Effects of immunomodulation on CD4^+^ T cells, CD4^+^CD25^+^Foxp3^+^ T_reg_ cells and T_regs_ activation markers in peripheral blood of treated hemophilia A inhibitor mice over time**. Lymphocytes were isolated from the blood of naive (light grid), inhibitor only (light slant), anti-CD20+FVIII (white), IL-2/IL-2mAb complexes+rapamycin+FVIII (light gray), IL-2/IL-2mAb complexes+anti-CD20+FVIII (dark gray), and IL-2/IL-2mAb complexes+rapamycin+anti-CD20+FVIII (black) treated mice. **(A)** CD4^+^ in total T cells and **(B)** CD4^+^CD25^+^Foxp3^+^ in CD4^+^ T cells were stained and analyzed by flow cytometry during the treatment. **(C)** Blood cells were also stained and analyzed for T_reg_ cells markers: CD25, GITR, and CTLA-4. Data shown are median fluorescence intensity (MFI) values of the three activation markers. Data shown is representative of two independent experiments.

Next, we evaluated changes in T- and B-cell populations of hemophilia A mice following treatment. We analyzed peripheral blood in mice treated with IL-2/IL-2mAb complexes+rapamycin+anti-CD20+FVIII (*n* = 4). Mice with neutralizing antibodies and those treated with anti-CD20+FVIII; IL-2/IL-2mAb complexes+rapamycin+FVIII; and IL-2/IL-2mAb complexes+anti-CD20+FVIII were used as control groups. Mice were treated weekly with the indicated regimen for 4 weeks. Flow cytometry analysis showed that the CD4^+^ T cells in total T-cell populations did not significantly change over time (Figure 4A). Interestingly, there was a slight decrease in the percentage and numbers of CD4^+^ T cells in anti-CD20 treated groups. However, the percentage of CD4^+^CD25^+^Foxp3^+^ T cells within the CD4^+^ T-cell compartment was significantly increased in the IL-2/IL-2mAb complexes treated groups compared to other groups for 4 weeks during treatment period (Figure 4B; *P* < 0.05). The expanded T_reg_ cells declined rapidly to baseline levels within 2 weeks post treatment. Similar to our previous studies (24, 25), IL-2/IL-2mAb complex-expanded T_reg_ cells showed considerably higher expression of molecules crucial for the suppressive function of T_reg_ cells, including CD25, glucocorticoid-induced tumor necrosis factor receptor (GITR), and cytotoxic T-lymphocyte antigen 4 (CTLA-4) (Figure 4C). The substantial increase in T_reg_ cells following injection of IL-2/IL-2mAb complexes occurred not only in blood but also appeared as a fivefold increase in spleen (Figure S2A in Supplementary Material; middle panel and Figure S2B in Supplementary Material; upper panel). Almost all expanded T_reg_ cells were Helios^+^ (Figure S2A in Supplementary Material; right panel and Figure S2B in Supplementary Material; upper panel) natural T_reg_ cells derived from thymus. In contrast, no significant change in the CD4^+^ T-cell population was observed in the spleen (Figure S2A in Supplementary Material; left panel and Figure S2B in Supplementary Material; upper panel). The expression levels of the activation markers of T_reg_ cells including CD25, GITR, and CTLA-4 after IL-2/IL-2mAb complex treatment also reached high levels in the spleen (Figure S2B in Supplementary Material; lower panel).

**Figure 5:**
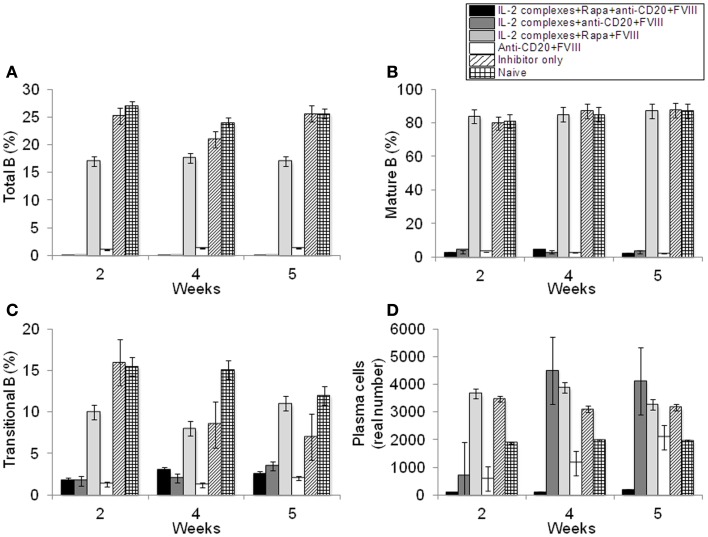
**Effects of immunomodulation on total B, mature B, transitional B, and plasma B-cells in peripheral blood of each mouse group**. Lymphocytes were isolated from the blood of naive (light grid), Inhibitor only (light slant), anti-CD20+FVIII (white), IL-2/IL-2mAb complexes+rapamycin+FVIII (light gray) IL-2/IL-2mAb complexes+anti-CD20+FVIII (dark gray), and IL-2/IL-2mAb complexes+rapamycin+anti-CD20+FVIII (black) treated mice. **(A)** B220^+^ B-cells, **(B)** IgM^+^IgD^hi^ B-cells, **(C)** transitional B-cells, and **(D)** plasma cells were stained and analyzed by flow cytometry during the treatment period (week 2, 4, and 5). Data shown are cell percentages **(A–C)** and real numbers **(D)** of the B-cell populations. Data shown is representative of two independent experiments.

Effects of anti-CD20 treatment were also evaluated on B-cell populations in the hemophilia A mice treated with combination therapy. After two treatments of anti-CD20, proportions of B-cell populations were measured by flow cytometry. We observed a significant decrease in proportions in the anti-CD20-treated mouse groups of total B-cells (B220^+^ cells) (Figure 5A and Figure S2C in Supplementary Material); mature B-cells (IgD^+^IgM^low^) (Figure 5B and Figure S2C in Supplementary Material); transitional B-cells (IgM^+^IgD^low^) (Figure 5C and Figure S2C in Supplementary Material); memory B-cells (IgM^−^IgD^−^) (Figure S2C in Supplementary Material); and plasma B-cells (B220^−^CD138^+^) (Figure 5D).

### Combination therapy depletes anti-FVIII-specific antibody-secreting cells (ASCs) and FVIII-specific memory B-cells in mice

**Figure 6:**
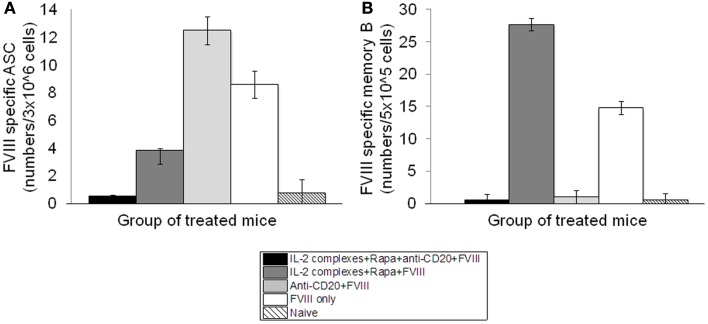
**Depletion of FVIII-specific ASCs (Antibody-Secreting Cells) and memory B-cells in the inhibitor mice treated with IL-2/IL-2mAb complexes plus rapamycin and anti-CD20**. Cells were isolated by MACS from spleens of naive (light slant), *FVIII* plasmid only (white), anti-CD20+FVIII (light gray), IL-2/IL-2mAb complexes+rapamycin+FVIII (dark gray), and IL-2/IL-2mAb complexes+rapamycin+anti-CD20+FVIII (black) treated mice (*n* = 2, each group) 2 weeks after treatment. **(A)** 3 × 10^6^ cells were used to detect FVIII-specific ASC cells by ELISPOT assay. **(B)** 5 × 10^5^ non-plasma cells were cultured and stimulated with FVIII at 10 U/ml for 6-days, and memory B-cells were detected by ELISPOT assay. Data shown are mean of spot numbers for each treated group (*n* = 2).

Hemophilia A mice were treated with IL-2/IL-2mAb complexes+ rapamycin+anti-CD20+FVIII; IL-2/IL-2mAb complexes+ rapamycin+FVIII; anti-CD20+FVIII; and FVIII alone as described previously. CD138^+^ cells were obtained from spleens of treated mice 2 weeks following treatment. Plasma cells were incubated with FVIII and analyzed for the formation of spots in an ELISPOT assay. Anti-FVIII ASCs correlated with the number of cells plated. Total anti-FVIII ASCs were reduced in mice treated with IL-2/IL-2mAb complexes+rapamycin+anti-CD20+FVIII and IL-2/IL-2mAb complexes+rapamycin+FVIII compared to other groups (Figure 6A). To confirm specificity of the assay, naive mice that had not been treated with FVIII were included. No FVIII-specific ASCs were detected in these mice (Figure 6A). Furthermore, no background staining in plates without FVIII immobilization was observed using cells obtained from FVIII-treated mice (data not shown). Evaluation of FVIII-specific ASCs and memory B-cells at later time points (4 and 6 weeks) after treatment showed similar results as those obtained at 2 weeks.

We also assessed whether depletion of FVIII-specific memory B-cells occurred following treatment. We isolated CD138^−^ spleen cells (presumably containing FVIII-specific memory B-cells) from hemophilia A mice treated with different single or combined regimens and re-stimulated these cells with high-dose FVIII (2 U/well). FVIII-specific memory B-cells were detected by ELISPOT. Interestingly, FVIII-specific memory B-cells were significantly reduced only in the IL-2/IL-2mAb complexes+rapamycin+anti-CD20+FVIII and anti-CD20+FVIII-treated mouse groups (Figure 6B).

## Discussion

Treatment of hemophilia A patients with inhibitors is very challenging and costly. In addition, probability of morbidity is increased in these patients. Although treatments with inhibitors has been successful in hemophilia mice, it is very challenging to decrease pre-existing anti-FVIII neutralizing antibodies in FVIII-primed hemophilia A mice. *In vivo* expansion of activated T_regs_ had profound suppressive effects and was able to prevent formation of anti-FVIII antibodies in both gene therapy and protein replacement therapy treated mice; however, this regimen can only transiently modulate pre-existing anti-FVIII immune responses. Anti-FVIII neutralizing antibodies can circulate for long periods and is thought to be partly due to the persistence of memory B and plasma cells. In a preliminary experiment, we found that either use of bortezomib (an inhibitor of 26S proteasome that can reduce/eliminate plasma cells) alone or in combination with T-cell-regulating agents such as IL-2/IL-2mAb complexes did not help reduce pre-existing neutralizing antibody titers (data not shown). Meslier et al. (29) reported similar results that bortezomib only delayed the onset of FVIII neutralizing antibodies in hemophilia A mice but failed to eliminate established anti-FVIII IgG-producing cells. Anti-CD20 mAbs can deplete pan B lymphocytes, from pre-B-cells to memory B-cells, ranging from 50 to 90%. Rituximab (anti-human CD20) is beneficial in treating patients with acquired hemophilia (30). Limited data have also been described in case reports with respect to the use of Rituximab in children (31, 32) and adults (31, 33) with congenital hemophilia A and neutralizing antibodies. It has been hypothesized that concurrent administration of anti-CD20 and high-dose FVIII might be beneficial to treat hemophilia A patients with neutralizing antibodies. Emerging data suggests that repopulating transitional murine and human B-cells (which increase markedly in numbers following anti-CD20 depletion therapy) exhibited potent regulatory activity (34). Therefore, use of anti-CD20 may promote effects distinct from merely reducing the mature B-cell pool. IL-10 expressing immature B-cells may promote a regulatory environment to aid in tolerance induction (35, 36). As anti-CD20 treatment exhibits a distinct mechanism of action and relatively few side effects, it is an excellent candidate agent for combinational approaches. Thus, we set out to study whether anti-CD20 treatment alone or in combination with other immune tolerance therapies can be more beneficial to treat hemophilia subjects with pre-existing inhibitory antibodies.

With anti-CD20 treatment alone, we found that anti-FVIII neutralizing antibody titers were reduced in both FVIII unprimed and primed hemophilia A mice. However, antibodies were not completely eliminated and FVIII activity did not improve in these animals. To further improve the therapeutic efficacy, we combined B-cell depletion using anti-CD20 with *in vivo* T_reg_ cell expansion using IL-2/IL-2mAb complexes+rapamycin to treat hemophilia A inhibitor mice. Our previous studies using IL-2/IL-2mAb complexes alone showed five to sevenfold expansion of highly suppressive T_reg_ cells *in vivo*, which induced long-term tolerance to FVIII in unprimed hemophilia A mice following gene therapy (24). IL-2/IL-2mAb complexes that can selectively promote human T_reg_ expansion are currently under development. Although no clinical trials have been initiated so far, IL-2/IL-2mAb complexes have high potential as a clinically feasible strategy; however, IL-2/IL-2mAb complex single treatment in the hemophilia A “inhibitor” mice only transiently reduced neutralizing antibody titers during treatment. It has been shown that rapamycin blocked T-cell-cycle progression from G1 to S phase after activation (37) and promoted TCR-induced T-cell anergy (38), achieving induction of operational tolerance (39). Additionally, rapamycin enriched antigen-specific Foxp3^+^ T_reg_ cells to promote organ transplant tolerance (40) and inhibited relapsing experimental autoimmune encephalomyelitis (EAE) by modulation of both effector/regulatory T cells (41). Moghimi et al. (42) reported that transient oral delivery of rapamycin combined with repeated injections of low doses of FVIII prevented induction of neutralizing antibody responses in hemophilia A mice. Thus, we adopted a treatment strategy that included rapamycin with IL-2/IL-2mAb complexes to enhance T_reg_ cell function *in vivo* for targeting T-cell-mediated immune responses and induction of FVIII-specific tolerance in hemophilia A mice with pre-existing antibodies.

Our results demonstrate that treatment with IL-2/IL-2mAb complexes+rapamycin had a synergistic effect when combined with anti-CD20 antibody to reduce neutralizing antibody titers. In contrast, mice treated with anti-CD20 only or different combinations only transiently reduced neutralizing antibody titers. In our immunomodulation studies, we also included weekly injection of FVIII protein (1 U/mouse/treatment) for induction of FVIII-specific tolerance. Furthermore, no increase in neutralizing antibody titers was observed following a second *FVIII* plasmid challenge, indicating induction of tolerance to FVIII. The mechanistic studies showed that the combined therapy promoted immune tolerance by increasing CD4^+^CD25^+^Foxp3^+^ T_reg_ cells and their activation markers CD25, GITR, and CTLA-4. Furthermore, combined treatment using anti-CD20 showed 98% depletion of total B, mature B, transitional B, and plasma cells. Changes in T/B-cell populations were not only detectable in the peripheral blood but also found in splenocytes isolated from treated mice. Most importantly, FVIII-specific ASCs and memory B-cells were reduced following treatment with combination therapy. It was demonstrated that anti-CD20 predominantly depleted FVIII-specific memory B-cells, and IL-2/IL-2mAb complexes+rapamycin helped reduce FVIII-specific ASCs. In Figure S2C in Supplementary Material, the percentage of total memory T cells (gated as IgM^−^IgD^−^ population) increased due to the more significant depletion (98%) of total B, mature B, and transitional B-cells. However, we found that FVIII-specific memory B-cells were significantly reduced after anti-CD20 treatment (shown in Figure 6B) in both mouse groups treated with anti-CD20 combination regimen. Whether the effects of IL-2/IL-2mAb complexes+rapamycin on B-cells were attributed to impaired T_H_ cell responses or directly mediated by the immune complexes is unclear. Upon second challenge with *FVIII* plasmid, the antibody titer remained low without an increase in FVIII activity, indicating that only partial tolerance against FVIII was achieved with combination therapy. Furthermore, treated mice were challenged with non-specific antigen, TNP-ficoll (24) at 16 weeks following treatment and responded similarly to control mice, demonstrating that this tolerance effect is FVIII-antigen-specific. Based on these results, we hypothesize that residual, long-lived FVIII-specific plasma cells may contribute to sustained low-titer neutralizing antibodies that limit the therapeutic benefits of immunomodulatory regimens.

Zhang et al. showed that a single dose of anti-CD20 IgG1 pretreatment prevented the increase of neutralizing antibodies in hemophilia A mice receiving high-dose protein replacement therapy (15). These antibodies can selectively deplete follicular B-cells while sparing marginal zone (MZ) B-cells as potential tolerogenic antigen-presenting cells. Interestingly, this treatment also led to an increase of T_reg_ cells. In a similar case, Sarikonda et al. showed that transient B-cell depletion with anti-CD20 IgG2a in combination with proinsulin resulted in modest increases in T_reg_ cells and offered limited efficacy in type 1 diabetes (T1D) prevention in NOD mice (7). In rhesus macaques, Mingozzi et al. (43) successfully used Rituximab in combination with cyclosporine to eradicate anti-human factor IX antibody following AAV8-mediated gene therapy. In addition, transient B-cell depletion by anti-CD20 IgG2a prevented FVIII inhibitor formation in hemophilia A mice receiving protein therapy but failed to induce long-term tolerance (16). In our study, administration of anti-murine CD20 IgG2a significantly reduced CD19^+^ B-cells in blood, spleen, and lymph nodes, as well as neutralizing antibody titers in FVIII plasmid-treated mice. Furthermore, our results showed that combination therapy targeting both B and T cells had better results to more significantly reduce anti-FVIII immune responses. As shown in our previous experiments, FVIII expression persisted in the liver for very long periods following hydrodynamic delivery of FVIII plasmids. With reversion of FVIII expression, FVIII antigen is present continuously during follow-up. Thus, it is concluded that long lasting FVIII-specific partial tolerance has been achieved with the combination therapy. In addition, viral gene transfer with adenoid-associated viral (AAV) vectors has been proposed as a therapeutic strategy for hemophilia A (44, 45). The immunomodulation regimens developed for transgene-specific immune responses may also be helpful in the design of modulatory protocols for immune responses to gene transfer vectors, in particular pre-existing immunity against viral vectors such as AAV. In addition, liver-directed gene transfers with several vectors have been associated with the induction of tolerance to the expressed transgene (46, 47). Combination of the immunomodulatory regimen developed in this study with liver gene transfer should increase the efficacy of tolerization against FVIII transgene. In summary, we demonstrated that IL-2/IL-2mAb complexes plus rapamycin acted synergistically with anti-CD20 to promote induction of immune tolerance to FVIII by increasing the number and function of CD4^+^CD25^+^Foxp3^+^ T_reg_ cells as well as eliminating both FVIII-specific ASCs and memory B-cells. These findings provide important preclinical evidence for the safety and enhanced therapeutic efficacy of the combined treatment for antibody responses in hemophilia A patients with pre-existing inhibitors.

## Materials and Methods

### Mice

All mice were kept according to the National Institutes of Health guidelines for animal care and the guidelines of Seattle Children’s Research Institute, and maintained at a specific pathogen-free (SPF) facility. Hemophilia A mice in a 129/SV × C57BL/6 mixed genetic background were generated by targeted disruption of exon 16 of *FVIII* gene (48) and were used at the age of 6–8 weeks.

### Immunomodulation using IL-2/IL-2mAb complexes in hemophilia a mice treating with FVIII plasmid

Hemophilia A mice were intravenously (i.v.) injected with 50 μg of *FVIII* plasmid [pBS-HCRHPI-FVIIIA (49)] in 2 ml phosphate-buffered saline (PBS) via tail vein in 8–10 s. IL-2/IL-2mAb complexes were prepared as previously described (17). One microgram recombinant mouse IL-2 (PeproTech, Rocky Hill, NJ, USA) was mixed with 5 μg anti-IL-2mAb (JES6-1A12) (eBioscience, San Diego, CA, USA), incubated at 37°C for 30 min, and then injected intraperitoneally (i.p.) into mice according to schedules specified in Results. Groups of IL-2/IL-2mAb complexes only treated mice, *FVIII* plasmid only treated mice, and naive mice were included as controls. Selected mice treated with immunomodulation received a second plasmid challenge at 9 weeks after the first treatment with immunomodulation therapy. Blood samples were taken from the retro-orbital plexus at serial time points and assessed for FVIII activity and anti-FVIII antibody levels.

### B-cells depletion by anti-CD20 treatment

Mice were depleted of B-cells using anti-murine CD20 IgG2a antibody (clone 18B12; Biogen Idec, Weston, MA, USA) at indicated schedules and dosages. To assess B-cell depletion, mice were given two i.v. doses 14 days apart, and peripheral blood and spleen tissues were collected at different time points after the first and second doses. Peripheral blood was collected in microcapillary tubes with 3.8% sodium citrate solution via retro-orbital plexus, centrifuged to remove plasma, and remaining cells suspended in PBS for staining and flow cytometry.

### Flow cytometry and antibodies

Cell suspensions of peripheral blood, lymph nodes (LNs from superficial cervical), and spleens of each treated mouse group were prepared according to standard protocols. Cell suspensions were stained for FACS analysis using the following antibodies (obtained from eBioscience unless otherwise stated): PE-Cy5-anti-mouse CD25; FITC-anti-mouse CD62L (L-selectin); Alexa Fluor^®^647-anti-mouse/rat Foxp3; PE-anti-mouse CD152 (CTLA-4); Alexa Fluor^®^700-anti-mouse CD4 (BD Pharmingen™; San Jose, CA, USA); PE-Cy7-anti-mouse GITR (BD Pharmingen™); Alexa Fluor^®^700-anti-mouse B220; FITC-anti-mouse IgD; PE-Cy7-anti-mouse IgM and PE-anti-mouse CD138. Cells were first stained for T-cell surface markers CD4, CD25, CD62L, and GITR, and subsequently stained intracellularly with T-cell markers for Foxp3 and CTLA-4 following the company protocol (eBioscience). For B-cell populations, cells were stained with surface markers B220, IgD, IgM, and CD138. Samples were analyzed on an LSRII flow cytometer (Becton Dickinson, Palo Alto, CA, USA) and data were analyzed using FlowJo software (Tree Star, Ashland, OR, USA).

### FVIII activities and inhibitor titers assays

Peripheral blood samples were taken from the experimental mice and collected in a 3.8% sodium citrate solution. FVIII activities were measured by a modified clotting assay using FVIII deficient plasma and reagents to measure activated partial thromboplastin time (APTT) and FVIII deficient plasma (49, 50). FVIII activities were calculated from a standard curve generated by using serially diluted normal human pooled plasma. Anti-FVIII activities were measured by Bethesda assay as previously described (51).

### ELISPOT assay

In preparation for the enzyme-linked immunospot (ELISPOT) assay, spleen cells from the treated mice were prepared to isolate the CD138^+^ (ASCs plasma) cells, 96-well filter plates (Millipore, MAHA N4510) were coated with the ASC cells (1 × 10^6^/well) and incubated overnight at 4°C. To detect the ASC cells, plates were washed and blocked with RPMI-1640 supplemented with 10% preselected fetal calf serum (Hyclone, Logan, Utah), 2 mM l-glutamine, 100 U/ml penicillin, 100 mg/ml streptomycin (all from Life Technologies), and 5 × 10^5^ M β-mercaptoethanol (Sigma-Aldrich) for 1 h at 37°C prior to detection. The restimulation of memory B-cells *in vitro* was achieved as described. (26) Briefly, spleen cells were isolated and depleted of CD138^+^ ASCs. CD138^−^ spleen cells were cultured at 3 × 10^5^ cells/well in RPMI-1640 medium at 37°C for 6-days. About 2 U/well of FVIII was added to the cells on day 0. After 6-days culture, newly formed ASCs were detected by ELISPOT assays.

## Author Contributions

Chao Lien Liu designed and performed research, analyzed data, and wrote the paper. Peiqing Ye performed research. Jacqueline Lin performed research and helped revise the manuscript. Chérie L. Butts provided anti-CD20 antibody and helped revise the manuscript. Carol H. Miao designed the project and research, analyzed data, and wrote the paper.

## Conflict of Interest Statement

The authors declare that the research was conducted in the absence of any commercial or financial relationships that could be construed as a potential conflict of interest.

## Supplementary Material

The Supplementary Material for this article can be found online at: http://www.frontiersin.org/journal/10.3389/fimmu.2013.00502/abstract

Figure S1 **B-cell depletion following anti-CD20 treatment in hemophilia A mice**. Mice were treated with i.v. injection of *FVIII* plasmid (50 μg at day 0) and anti-CD20 (gray) or IgG2a isotype control (white) at a dose of 250 μg/injection at days 0 and 14. PBMCs and spleen cells isolated from anti-CD20 treated hemophilia A mice were stained with FITC-CD19, and APC-B220 at 0.5, 2, 4, 8, 12, and 16 weeks following plasmid treatment and analyzed by flow cytometry. Naïve (black) and IgG control-treated hemophilia A mice were used as controls. **(A)** Representative plot for blood cells at different time points, **(B)** Total B-cell (CD19^+^B220^+^) depletion in blood over time, **(C)** Total B-cell (CD19^+^B220^+^) depletion in spleen over time. Data shown is representative of two independent experiments.

Figure S2 **Effects of immunomodulation on both T and B-cells isolated from spleens of each treated mouse group**. Spleen **(A–C)** cells were collected and isolated at serial time points from naive (light slant), *FVIII* plasmid only (white), anti-CD20+FVIII (light gray), IL-2/IL-2mAb complexes+rapamycin+FVIII (dark gray), and IL-2/IL-2mAb complexes+rapamycin+anti-CD20+*FVIII* (black) treated mice (*n* = 2, each group). Cells were stained and analyzed for T-cell populations **(A,B)** and B-cell populations **(C)**. Data shown is representative of two independent experiments.
