# Supplementary material for: Anti-CD20 as the B-Cell Targeting Agent in a Combined Therapy to Modulate Anti-Factor VIII Immune Responses in Hemophilia a Inhibitor Mice
Source: Front Immunol. 2014 Jan 6;4:502. doi: 10.3389/fimmu.2013.00502 (PMC3881000; doi:10.3389/fimmu.2013.00502)
Supplement: Figure S1 — B-cell depletion following anti-CD20 treatment in hemophilia A mice. Mice were treated with i.v. injection of FVIII plasmid (50 μg at day 0) and anti-CD20 (gray) or IgG2a isotype control (white) at a dose of 250 μg/injection at days 0 and 14. PBMCs and spleen cells isolated from anti-CD20 treated hemophilia A mice were stained with FITC-CD19, and APC-B220 at 0.5, 2, 4, 8, 12, and 16 weeks following plasmid treatment and analyzed by flow cytometry. Naïve (black) and IgG control-treated hemophilia A mice were used as controls. (A) Representative plot for blood cells at different time points, (B) Total B-cell (CD19+B220+) depletion in blood over time, (C) Total B-cell (CD19+B220+) depletion in spleen over time. Data shown is representative of two independent experiments. [file 71492_Miao_Presentation1.PDF]

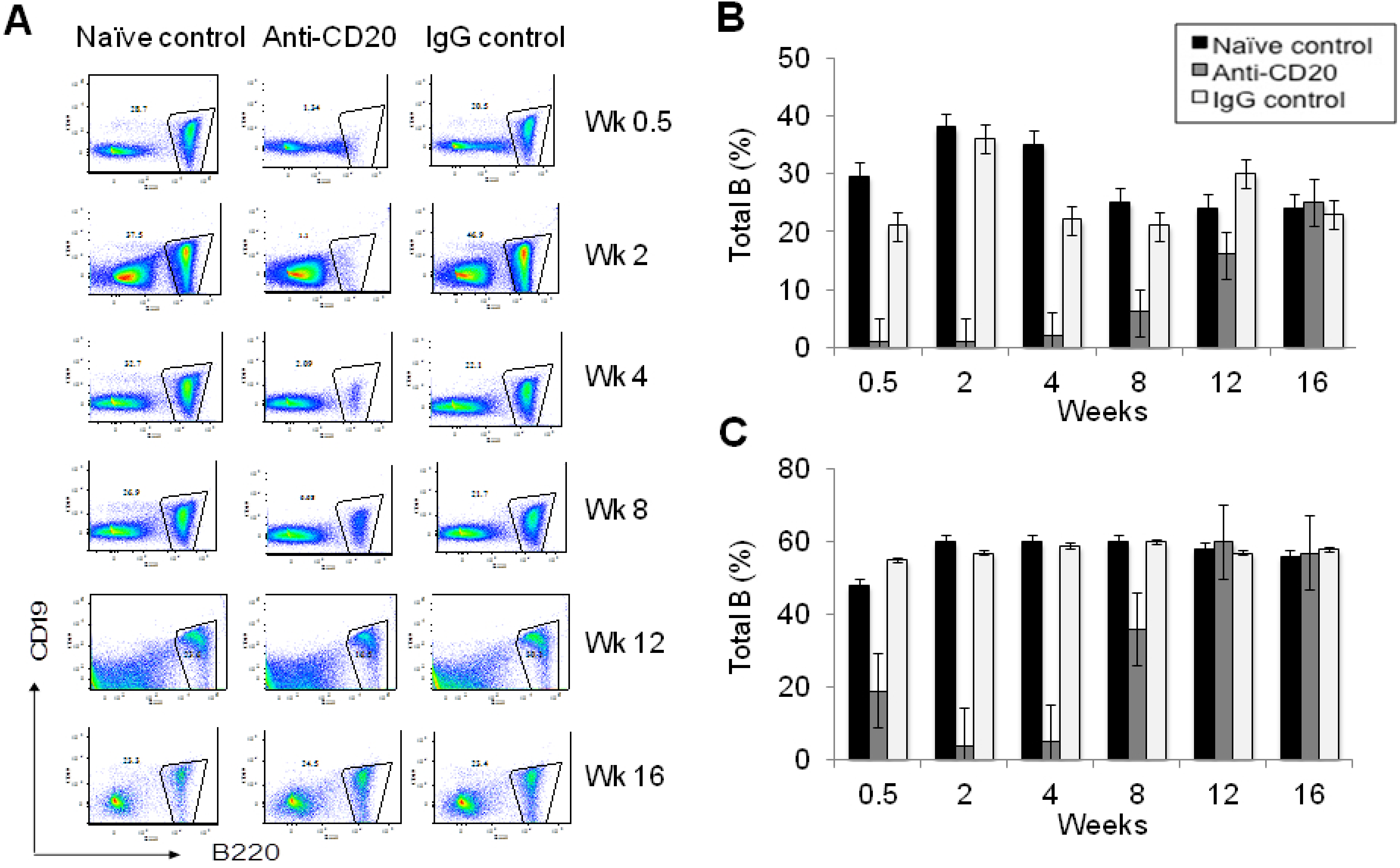

Supplemental 1. B cell depletion following anti-CD20 treatment in hemophilia A mice. Mice were treated with i.v. injection of *FVIII* plasmid (50 µg at day 0) and anti-CD20 or IgG2a isotype control at a dose of 250 µg/injection at days 0 and 14. PBMCs and spleen cells isolated from anti-CD20 treated hemophilia A mice were stained with FITC-CD19, and APC-B220 at 0.5, 2, 4, 8, 12, and 16 weeks following plasmid treatment and analyzed by flow cytometry. Naive and IgG control-treated hemophilia A mice were used as controls. (A) Representative plot for blood cells at different time points, (B) Total B cell ($CD19^+B220^+$) depletion in blood over time, (C) Total B cell ($CD19^+B220^+$) depletion in spleen over time. Data shown is representative of two independent experiments.

Supplemental. 1
