# Supplementary material for: Anti-CD20 as the B-Cell Targeting Agent in a Combined Therapy to Modulate Anti-Factor VIII Immune Responses in Hemophilia a Inhibitor Mice
Source: Front Immunol. 2014 Jan 6;4:502. doi: 10.3389/fimmu.2013.00502 (PMC3881000; doi:10.3389/fimmu.2013.00502)
Supplement: Figure S2 — Effects of immunomodulation on both T and B-cells isolated from spleens of each treated mouse group. Spleen (A–C) cells were collected and isolated at serial time points from naive (light slant), FVIII plasmid only (white), anti-CD20+FVIII (light gray), IL-2/IL-2mAb complexes+rapamycin+FVIII (dark gray), and IL-2/IL-2mAb complexes+rapamycin+anti-CD20+FVIII (black) treated mice (n = 2, each group). Cells were stained and analyzed for T-cell populations (A,B) and B-cell populations (C). Data shown is representative of two independent experiments. [file 71492_Miao_Presentation2.PDF]

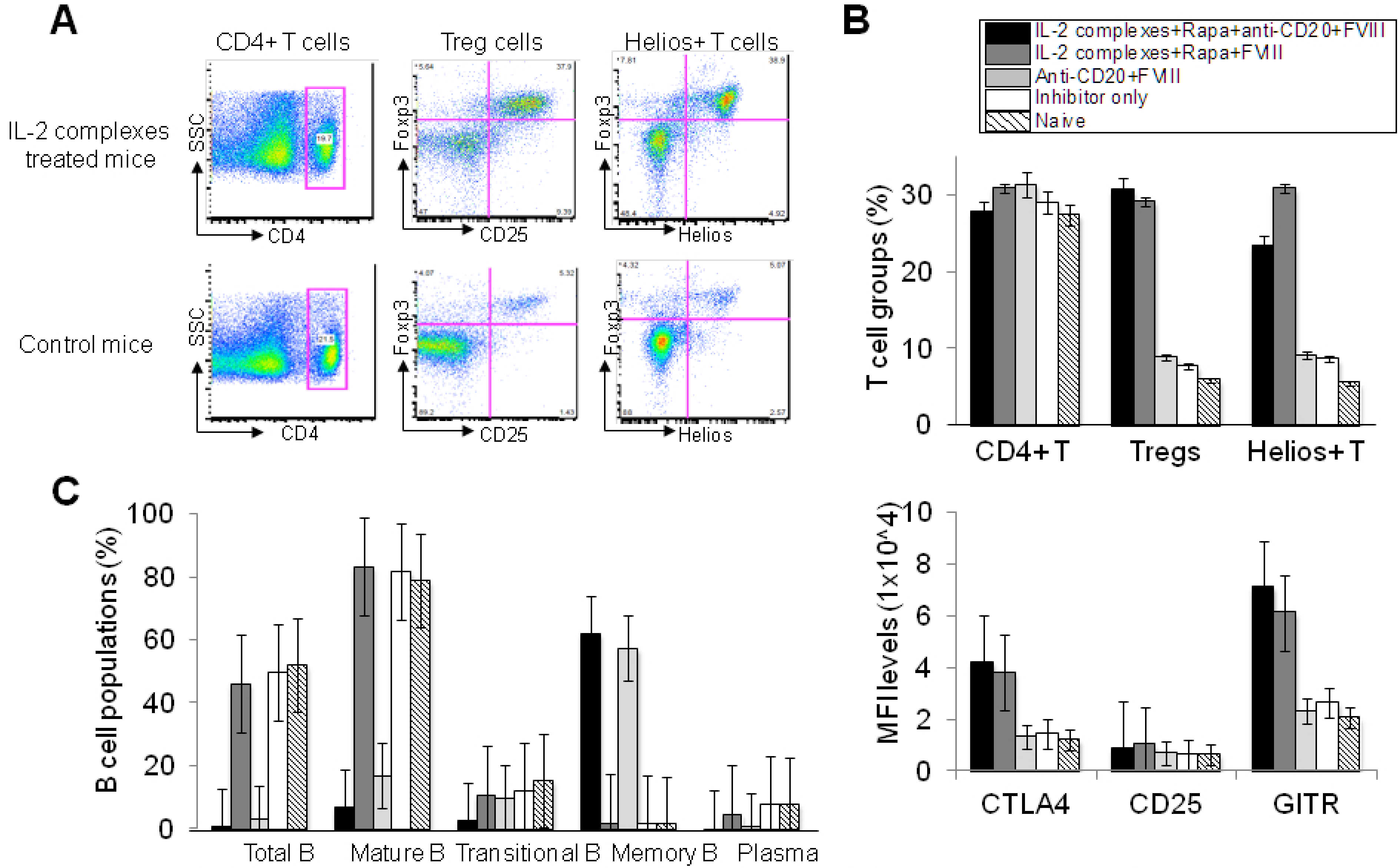


**Supplemental 2. Effects of immunomodulation on both T and B cells isolated from spleens of each treated mouse group.** Spleen (A, B and C) cells were collected and isolated at serial time points from naive (light slant), *FVIII* plasmid only (white), anti-CD20 + FVIII (light gray), IL-2/IL-2mAb complexes + rapamycin + FVIII (dark gray) and IL-2/IL-2mAb complexes + rapamycin + anti-CD20 + *FVIII* (black) treated mice (n=2, each group). Cells were stained and analyzed for T cell populations (A, B) and B cell populations (C). Data shown is representative of two independent experiments.

## Supplemental. 2
